# COVID-19, Isolation, Quarantine: On the Efficacy of Internet-Based Eye Movement Desensitization and Reprocessing (EMDR) and Cognitive-Behavioral Therapy (CBT) for Ongoing Trauma

**DOI:** 10.3390/brainsci11050579

**Published:** 2021-04-30

**Authors:** Rinaldo Livio Perri, Paola Castelli, Cecilia La Rosa, Teresa Zucchi, Antonio Onofri

**Affiliations:** 1 Faculty of Psychology, University Niccolò Cusano, Via Don Carlo Gnocchi 3, 00166 Rome, Italy; 2 De Sanctis Clinical Center, 00166 Rome, Italy; paola.castelligattinara@gmail.com (P.C.); larosacecilia@gmail.com (C.L.R.); dottoronofri@gmail.com (A.O.); 3 School of Psychotherapy, Psicoterapia Training School (PTS), 00166 Rome, Italy; 4 Zucchi Psychiatry and Psychotherapy Office, Private Practice, Via Giuseppe Catani, 28/C, 59100 Prato, Italy; zucchiteresa@gmail.com

**Keywords:** EMDR, CBT, COVID-19, psychotherapy, online therapy, trauma

## Abstract

Literature points to cognitive-behavioural therapy (CBT) and eye movement desensitization and reprocessing (EMDR) as evidence-based therapies for trauma-related disorders. Treatments are typically administered in a vis-à-vis setting with patients reporting symptoms of a previously experienced trauma. Conversely, online-therapies and ongoing trauma have not received adequate attention. This study aimed to compare the efficacy of two brief treatments for health professionals and individuals suffering from the circumstances imposed by the coronavirus disease 2019 (COVID-19) pandemic. The EMDR and the trauma focused-CBT were administered online during the earliest stage of distress to manage the ongoing trauma associated to quarantine or disease. Thirty-eight patients satisfying the Diagnostic and Statistical Manual of Mental Disorders (DSM-5) criteria for acute stress disorder were randomly assigned to the EMDR or CBT treatment. Both groups received a 7-session therapy, and psychometric tests were administered before, after the treatment and at one-month follow-up to assess traumatic symptoms, depression and anxiety. Results revealed that both treatments reduced anxiety by 30%, and traumatic and depressive symptoms by 55%. Present findings indicate the internet-based EMDR and CBT as equally effective brief treatments, also suggesting a maintenance of the effects as indicated by the follow-up evaluation. The EMDR and CBT might be considered as first line therapies to treat the ongoing trauma and to prevent the sensitization and accumulation of trauma memories.

## 1. Introduction

In the last few decades, an extensive literature has recommended eye movement desensitization and reprocessing (EMDR) therapy as a psychotherapeutic intervention for anxiety disorders (for reviews see [1,2,3]). In particular, EMDR is mostly provided for post-traumatic stress disorder (PTSD; see [4] for a review), for which the trauma-focused cognitive-behavioural therapy (TF-CBT) has been proven to be a first-line treatment as well [5]. Nowadays, the efficacy of both the EMDR and CBT for traumatic disorders is unanimously considered superior to waiting list or other therapies (for a meta-analysis see [6]), while the direct comparisons of these approaches yielded conflicting results. In fact, some investigations reported the CBT for trauma to be more effective than EMDR [7,8], whereas others concluded the opposite [9,10,11,12]. However, it is noteworthy that a few recent meta-analyses have directly addressed this issue suggesting that TF-CBT produces the strongest evidence for recent trauma [13], while CBT and EMDR are equally efficacious for PTSD [14,15] and complex PTSD symptoms in the adult population [16].

Nevertheless, research has almost entirely focused on treatments of established PTSD, while early psychological interventions for the recent trauma have mostly been neglected [17,18]. Likewise, only a few investigations focused on ongoing trauma, and mostly in the childhood population [19,20,21,22] or refugees [23,24]. The question of if and when to intervene during a traumatic experience is crucial for mental health professionals as it has been documented that among individuals exposed to trauma one third remain symptomatic for 3 or more years with greater risk for secondary complications [25]. However, it also needs to be recognized that ongoing trauma could sometimes hinder patients’ access to care making the psychotherapeutic intervention even more problematic. In such situations, internet-based therapies might reflect the only way to treat trauma-exposed patients: this was the case of the strict Italian lockdown during the initial and more dramatic phase of the coronavirus disease 2019 (COVID-19) pandemic. As for the online modality, it is worth noting that an extensive meta-analysis revealed that its effectiveness is quite similar to traditional face-to-face psychological interventions [26].

The present study aimed to compare the efficacy of two early psychotherapeutic interventions for Italian health professionals and individuals suffering from the circumstances imposed by COVID-19 pandemic. To this goal, the EMDR and the TF-CBT were provided online to manage the ongoing trauma associated with quarantine, isolation or work in COVID-19 hospital wards. The decision to provide remote therapeutic support stemmed from the prohibition of physical contact, such as from the need to offer support as early as possible to reduce acute distress and prevent the sensitization and accumulation of trauma memories. In particular, patients requiring psychological support were invited to a first clinical interview, screened for traumatic, anxiety and depression symptoms, and only those who satisfied DSM-5 criteria for acute stress disorder (ASD) were randomly assigned to EMDR or TF-CBT group. Both groups received a 7-session therapy for a total duration of about 3 weeks (2 sessions per week). Follow-up measures were collected to assess the maintenance of the effects after the treatment, and both treatments were based on established protocols (see Methods section) to provide mental health professionals with practical guidance to stabilize trauma-risk patients.

Since, to the best of our knowledge, no studies are available on the comparison of these two treatments for ongoing trauma, we rely on the meta-analysis of Lewis and colleagues [14] on the established trauma to predict an equal efficacy of the two approaches on the observed measures. In particular, we expected a significant and stable reduction of traumatic, anxiety and depressive symptoms in both EMDR and TF-CBT groups

## 2. Materials and Methods

### 2.1. Participants

Forty two subjects requiring psychological support to manage the ongoing trauma associated with quarantine, isolation or work in COVID-19 hospital wards took part in the study. They were recruited from physician referrals, advertisements on internet and media, and from the extensive network of health and psychological associations collaborating with the promoter organizations. After the first session and clinical assessment, patients were considered eligible for the study (and admitted to the second session) if they met the following criteria: (I) aged between 18 and 65 years; (II) able to satisfy DSM-5 criteria for acute stress disorder; (III) not involved in psychopharmacological or psychological therapy outside the present study; (IV) absence of substance addiction, psychotic disorders, severe depression with suicidal proposal. After initial screening 4 subjects were excluded from the study and the remaining 38 patients were randomly assigned to EMDR (*n* = 19, 5 males, age = 48.3 ± 13.6) and TF-CBT protocol (*n* = 19, 6 males, age = 52.4 ± 10.6). The study was conducted in accordance with the ethical standards of the 1964 Declaration of Helsinki and received the EMDR Europe Ethical Approval. All patients provided informed consent when participating in the study. 

### 2.2. Procedure and Treatments

Patients were randomly assigned to EMDR Recent Traumatic Episode Protocol (R-TEP [18]) or TF-CBT group and invited to the first online session on the Skype platform: they were informed in detail about procedures and asked to give informed consent that was video recorded. After that, patients received a link to a reserved web page for tests compilation: results were transmitted to the first author of the study who was responsible for scoring, data storage and privacy protection.

Therapies were dispensed online by 14 experienced psychotherapists (more than 10 years of certified clinical practice): 8 for the EMDR group and 6 for the TF-CBT group. All therapists were constantly supervised by author A.O. Both groups received a 7-sessions therapy: 2 sessions per week for a total duration of about 3 weeks (see Table 1 for the detailed description of each session in the two treatments). After the last session and at one month follow-up patients were asked to re-compile the same tests administered during the assessment stage (see the Measures and analyses for details).

### 2.3. Measures and Analyses

The following self-rated tests were administered three times (pre-treatment, post-treatment and at 1 month follow-up) to measure the main dependent variables of the study:

*PTSD checklist for DSM-V* (PCL-5 [27]): this 20-item measure assesses the DSM-5 symptoms of PTSD. Participants rated each item on a five-point Likert scale, from “Not at all” (0) to “Extremely” (4). Total score ranges from 0 to 80 with higher scores reflecting greater severity of traumatic symptoms. High internal consistency (Cronbach’s α = 0.92) was demonstrated for the present sample. 

*State Trait Anxiety Inventory* (STAI-Y1 [28]): this 20-item inventory measures the state anxiety. Participants rated each item using a four-point Likert scale, from “Not at all” (1) to “Very much so” (4). Total score ranges from 20 to 80 with higher scores indicating greater state anxiety. High internal consistency (Cronbach’s α = 0.87) was demonstrated for the present sample. 

*Beck Depression Inventory-II* (BDI-II [29]): 21-item self-report measure of depression. Each item is rated on a 4-point Likert-type scale ranging from 0 to 3, with higher scores indicating higher levels of depression. Total score ranges from 0 to 63 with higher scores indicating greater level of depression. High internal consistency (Cronbach’s α = 0.91) was demonstrated for the present sample.

Scores of the above reported tests were submitted to 2 × 3 repeated measures analyses of variance (RM-ANOVAs) with therapy (EMDR, TR-CBT) and Time (pre-treatment, post-treatment, follow-up) as between- and within-subjects variable respectively. Further, in order to confirm groups homogeneity at baseline, demographic data and pre-treatment scores were statistically compared between EMDR and TF-CBT groups (independent samples t-test). The overall alpha level was fixed at 0.05, and significant results corrected using Bonferroni test for multiple comparisons. Effect size was measured with the partial eta squared (η^2^p): according to Cohen [30], η^2^p ≥ 0.01 was interpreted as a small effect, ≥0.06 as a moderate effect, and ≥0.14 as a large effect.

## 3. Results

**Figure 1:**
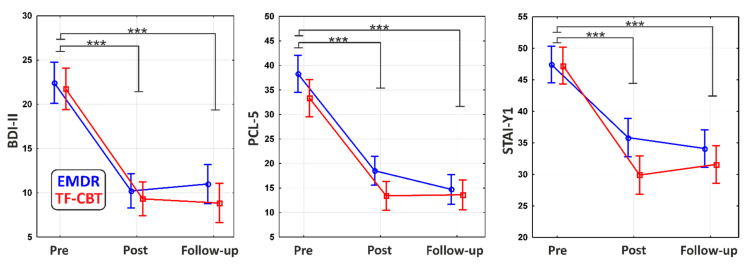
Mean scores of the Beck Depression Inventory-II (BDI-II, **left**), post-traumatic stress disorder checklist for DSM-V (PCL-5, **middle**) and State Trait Anxiety Inventory (STAI-Y1, **right**) tests in the three time points for the EMDR (blue) and TF-CBT (red) groups. Vertical bars denote standard error of the means. *** *p* < 0.0001.

No age difference emerged between EMDR and TF-CBT (*t* = 0.6, *p* > 0.05); also, the pre-treatment scores of the two groups did not differ for the BDI-II (*t* = 0.2, *p* > 0.05), PCL-5 (*t* = 0.9, *p* > 0.05) and STAI-Y1 (*t* = 0.03, *p* > 0.05) indicating a condition of demographic and clinical homogeneity between patients of the two treatments. As a further confirmation, the RM-ANOVAs effect of therapy and therapy × time interaction did not reach statistical significance (all ps > 0.05), indicating that no treatment was superior to the other, and that the scores of the two groups were similar at all time points for all psychological tests. On the contrary, RM-ANOVAs yielded a significant main effect of time for the PCL-5 (F_2,72_ = 57.12, *p* < 0.0001, η^2^p = 0.61), STAI Y-1 (F_2,72_ = 41.75, *p* < 0.0001, η^2^p = 0.53) and BDI-II scores (F_2,72_ = 50.17, *p* < 0.0001, η^2^p = 0.58). Post-hoc comparisons are reported in Figure 1 showing the scores of both groups for the three tests in the different time points: the graph reveals similar values of EMDR and TF-CBT for all the considered measures, and a significant score decrease from pre- to post-treatment, and from pre-treatment to follow-up for both groups (all Bonferroni-corrected ps < 0.0001). A summary of all the considered values is reported in Table 2.

Although preliminary *t*-tests ruled out any group-difference at pre-treatment, we decided to carry out further control analyses by performing separate analyses of covariance (ANCOVAs) with the initial score as the covariate, and the post-treatment and follow-up score as the dependent variable. For all the three psychological measures, results did not reveal significant effects of therapy, time and therapy × time (all ps > 0.05). These data corroborated previous findings indicating that, even when removing any possible variance of pre-treatment, EMDR and TF-CBT yielded to similar scores at post-treatment, and that these remained unchanged at follow-up.

## 4. Discussion

The present study aimed at evaluating the efficacy of two brief psychological interventions for people exposed to traumatic-like experiences associated to the Italian first stage of the COVID-19 pandemic. To the best of our knowledge, this is the first investigation where the TF-CBT and the EMDR were randomly administered and compared as ongoing trauma therapies. Moreover, the online modality represents a further element of novelty compared to the previous studies in this field. The main findings revealed that the brief EMDR and TF-CBT were equally efficacious, and both yielded a relevant improvement on the outcome measures. In particular, after the 7-session treatment, state anxiety decreased by about 30% while the traumatic and the depressive symptoms were reduced by about 55%, in line with previous investigations showing that PTSD treatments were also associated with reductions in depressive symptoms (see [6] for a meta-analysis). These results were confirmed at 1-month follow-up where traumatic symptoms reduced by an additional 11%: even if not statistically significant, these follow-up data are relevant to the treatment target and its stability over time.

Providing early psychological interventions for trauma is crucial to prevent the consolidation of traumatic memories [17,18], such as the psychiatric conditions that can develop comorbid with the trauma after an ASD [31]. However, the lack of solid literature on the ongoing trauma in the adult population left open the question of what intervention to propose: on this point, present results are consistent with the extensive evidence on consolidated trauma and PTSD that documented equal efficacy of EMDR and CBT approaches in the face-to-face setting [14]. Furthermore, the fact that the current findings come from online-therapies suggests that the two treatments are both convertible to internet mode (see [26] for similar conclusions), and therefore recommended for early interventions on the ongoing trauma. Indeed, beyond the pandemic, remote therapy may be needed for several conditions that hinder physical contacts, such as the extreme level of distress associated with leaving home for some patients, earthquakes that make a place unsafe, or the many situations that may limit the clients’ access to care (e.g., unavailability of a specialized therapist nearby).

A limitation of the present investigation might consist in the active-active comparison design, that is the absence of a waiting list as in most of the studies in this field [15]. However, we must consider that traumatic symptoms tend to persist in the absence of treatment [6] and therefore the changes at post-treatment are unlikely to be due to the passage of time [8]. Moreover, the short duration of the two treatments (i.e., 3 weeks) together with the persistence of the COVID-19 circumstances further excludes the alternative hypothesis of spontaneous remission in all patients. In fact, it is noteworthy that the subjects of the present study were recruited during the first Italian lockdown (from March to May 2020) that presented some key features: Italy was the only “red” country outside Asia, and the virus scared people more because of its novelty, the absence of diagnostic tools and therapies, and therefore the impressive mortality among the newly affected. All these conditions allowed the traumatic conditions to remain ongoing, even at the end and after treatment. As for the follow-up, we recognize that the 1-month assessment may limit monitoring of medium- and long-term outcomes, thus future studies should also consider multiple or delayed evaluations.

The possibility to provide these therapies online could indirectly contribute to the debate on the mechanisms underlying EMDR therapy. Despite the increasing number of studies published in recent years about the utility of the eye movements and bilateral stimulation in the EMDR practice [32,33] it is not possible to establish firm conclusions. As these patients have self-administered the hand-tapping, this raises questions about the rationale of guided ocular movements that some empirical investigations considered unnecessary (see [15,34] for me-ta-analyses). Nevertheless, we must consider that the specific point about usefulness of bilateral stimulation in EMDR remain controversial (see [35] for a review) and that recent prominent studies have suggested new hypothesis about underlying neuro-physiological mechanisms [36,37]: this topic is, however, outside the scope of the present study and probably deserves a comparison with a no-stimulation condition to be tested.

In conclusion, we might suggest that internet-based EMDR and TF-CBT were equally effective for ongoing trauma and related symptoms as they both adopted exposure procedures whose rationale is well-known for trauma-therapy [38]. Furthermore, as the two treatments were based on specific protocols (see Methods), the present findings are open to the possibility of adopting these brief online-therapies as a first line treatment for traumatic conditions that require remote and early interventions, in line with recommendations for established PTSD and face-to-face settings [14]. Future studies are needed to directly test the preventive capacity of these interventions with respect to long-term consolidation of traumatic memories and associated psychiatric disorders in order to provide mental health professionals with evidence-based guidelines for managing trauma.

## Figures and Tables

**Table 1:** Eye movement desensitization and reprocessing (EMDR) and trauma-focused cognitive-behavioural therapy (TF-CBT) protocols: activities planned in each of the 7 sessions.

Session	EMDR	TF-CBT
1	General anamnesis; presentation of the intervention; instructions and invitation to fill in the online assessment	General anamnesis; presentation of the intervention; instructions and invitation to fill in the online assessment
2	Trauma psychoeducation; sleep quality and eating monitoring; “four elements” and “safe place” exercise; homework: to practice the exercise 2–3 times a day	Trauma psychoeducation; sleep quality and eating monitoring; “four elements” and “safe place” exercise; breathing retraining and grounding; homework: to practice the exercise 2–3 times a day
3	Stabilization exercises. Training on the relational and mastery skills	Stabilization exercises. Jacobson’s Progressive muscle relaxation; homework: practice relaxation, grounding and breathing retraining every day
4	Recent events protocol; Exposure with self-tapping. Identifying and desensitizing first Points of Disturbance (PoD) with Bilateral Stimulation (Tapping or Butterfly Hug)	Traumatic events/situations: prolonged verbal exposure and cognitive restructuring; identifying the most disturbing avoidance behaviour to deal with in vivo exposure; brief mindfulness training; homework: listening the recorded session, practice the relaxation, breathing retraining or safe place exercise after each exposure
5	PoDs identification and desensitization with Bilateral Stimulation (part 1)	
6	PoDs identification and desensitization with Bilateral Stimulation (part 2)	
7	Review and closure	Review and closure

**Table 2:** Scores of the three psychological tests at pre-treatment, post-treatment and follow-up for the EMDR and TF-CBT groups. Values are mean (SD).

	EMDR			TF-CBT		
	Pre	Post	Follow-Up	Pre	Post	Follow-Up
BDI-II	22.4 (10.5)	10.2 (6.4)	11 (9.3)	21.7 (9.6)	9.3 (9.9)	8.8 (10.1)
PCL-5	38.2 (16.7)	18.5 (12.3)	14.7 (13.7)	33.3 (16.2)	13.4 (12.9)	13.6 (12.5)
STAY-Y1	47.4 (13.1)	35.8 (14.5)	34.1 (14.9)	47.2 (12.2)	29.8 (11.8)	31.5 (10.6)

## Data Availability

The data presented in this study are available on request from the corresponding author.
